# Traits of cancer patients and CAM usage

**DOI:** 10.1007/s00432-021-03605-7

**Published:** 2021-04-02

**Authors:** Sabine Andrea Dufter, Jutta Hübner, Emadaldin Ahmadi, Bijan Zomorodbakhsch

**Affiliations:** 1 Innstraße 3, 83278 Traunstein, Germany; 2 Klinik Für Innere Medizin II, Am Klinikum 1, 07747 Jena, Germany; 3 Onkologische Kooperation Harz, Goslar, Germany

**Keywords:** CAM, Personal traits, Cancer, Integrative medicine, Spirituality, Attentiveness

## Abstract

**Background:**

The use of Complementary Alternative Medicine (CAM) Methods is increasing and therefore gaining importance also in conventional western medicine. Identifying personal traits to make out by whom and why CAM is used can help physicians in successful physician–patient interaction, and thus improve patient’s compliance and trust towards their physician.

**Patients and methods:**

A questionnaire was passed on to cancer patients in an ambulant clinical and a rehabilitation setting. Multiple regression analyses were run to examine possible predictors for CAM use, such as gender, age, level of education, spirituality, attentiveness, self-efficacy and resilience. To differentiate within CAM users, two dependent variables were created: “holistic and mind–body methods”, such as Yoga, meditation or Homeopathy and “material based methods”, such as food supplements or vitamins.

**Results:**

Higher level of education, younger age and religion-independent attentiveness were significant predictors for the use of “material based methods”. Female gender, higher education and religious spirituality were detected as significant predictors for “holistic and mind–body methods”.

**Conclusion:**

This study is among the first to take a more detailed look at how numerous personal traits are associated with the use of CAM methods and differentiate between the applied methods. Our finding should be considered by conventional health care providers and could be integrated into a holistic assessment, to offer information about complementary medicine and meeting patients’ needs.

## Introduction

### CAM—definition and usage

Complementary medicine refers to a heterogeneous group of therapies that fall traditionally beyond the range of conventional medicine, but can be used alongside conventional treatment. In severe diseases that require aggressive therapies, such as chemotherapy in cancer treatment, complementary medicine can support the patients’ well-being and compliance. It can contribute to a more wholesome approach in patient care and meeting patients’ demands that cannot be satisfied by conventional medicine (Ernst 2000).

A study in 2011 showed that 66.5% of cancer survivors state to have used complementary medicine alongside the conventional treatment of their disease (Mao et al. 2011). Furthermore, meta-analyses have shown that CAM use has been increasing in the last decades (Frass et al. 2012). This shows the growing importance of integrating CAM into traditional health care structures.

CAM ranges from non-material methods, such as prayer, massage, music or meditation to material methods, such as vitamin supplements, homeopathy, Chinese teas and many more (Kang et al. 2014). It was found that especially biologically based therapies, relaxation techniques, prayer and meditation were the most frequently applied methods among participants of a German online survey in 2014 (Huebner et al. 2014).

Research indicates that the wide range of CAM methods are used for different reasons (Huebner et al. 2014). Whereas prayer, meditation and music were found to be used in the intention to maintain a feeling of control over life, other typical aims of using CAM were immune enhancement and pain control (Mao et al. 2011; Kang et al. 2014).

### Traits associated with CAM use

It has been shown in various studies that women, and patients with a higher level of education tend to be the typical CAM users among cancer patients (Molassiotis et al. 2005; Frass et al. 2012; Dubois et al. 2019). Nevertheless, only few studies have identified other characteristics that are significantly associated with CAM use in cancer patients. Research in the general population has shown that health behaviors, spirituality and openness are strong predictors of CAM use (Thomson et al. 2014; Dessio et al. 2004).

Spirituality and the use of CAM could be identified as two associated concepts in various studies (Trinkaus et al. 2011; James and Bah 2014; Ellison et al. 2012). They stated that spirituality could be a good predictor for CAM use. Spirituality and spiritual needs cannot always be approached with religiousness, instead should be treated as two concepts, independent from each other (Thoresen and Harris 2002). The necessity for meeting patients’ spiritual needs also in a non-religious way, especially for those patients who lack a religious community to turn to, has been highlighted. Studies have shown that spiritual requirements are often not satisfied by conventional medicine (Balboni et al. 2007), but can be met in an appropriate way by CAM methods (Hsiao et al. 2008).

Research indicated that spirituality is positively correlated with an active coping style, quality of life and well-being in cancer patients (Holland et al. 1999; Peterman et al. 2002; Trinkaus et al. 2011). These findings provided convincing intension to encourage patients in their spiritual requirements. Measuring and approaching the spiritual needs of cancer patients with non-religious CAM methods, such as meditation, could support compliance and meeting patients’ holistic needs during cancer treatment in a safe way.

Attentiveness is a concept that can be perceived as a character trait; it can be enhanced by training, for example by meditation or prayer (Zale et al. 2018). Attentiveness is traditionally an essential teaching in ancient Asian religions, such as Buddhism (Baltzell and Cote 2017). The last decades show a surge in mindfulness research in Western societies with a focus on the attempt to define the concept without integrating pre-existing religious doctrines. A definition by Brown and Ryan (2003) described mindfulness as the ability to be in the present, paying attention to oneself and the moment. It has been established in various studies that mindfulness promotes well-being and health (Baer 2003; Creswell et al. 2019). Furthermore, it has been investigated as a predictor for social behavior (Lakey et al. 2007) However, the relation between CAM use and mindfulness is poorly studied.

Self-efficacy is comparable with positive self-esteem, the ability to find solutions for personal challenges within oneself (Flammer 2015). Self-efficacy is positively correlated with health behavior (Strecher et al. 1986). Interestingly self-efficacy could be enhanced by training methods and plays a critical role in behavioral therapy (Bandura 2004). In this context, it seems reasonable to consider self-efficacy as a superior predictor of CAM use.

Resilience is a concept that has been defined in various ways. This might be an explanation of why measuring resilience can be challenging (Rosenberg et al. 2013). An important principle of the definition of resilience is the ability to recover from adversities, such as severe disease (Cosco et al. 2016). Scores to measure resilience have been proven to be valid (Cosco et al. 2016), and the association between resilience and the use of alternative methods has been a field of interest (Davidson et al. 2005). Nevertheless, research using resilience as a predictor for CAM usage is still sparse.

The interest has risen in identifying more predictors for CAM use in cancer patients, to provide better patient centered care. Focusing on the patient’s personal traits, less tangible characteristics could gain relevance.

Identifying the personal characteristics of cancer patients can help predict interest in CAM and CAM use. This can be relevant for health care providers to meet patient’s needs for the information, prevent negative interactions between conventional and complementary therapies and improve compliance. The aim was to identify traits, such as spirituality, resilience, attentiveness and self-efficacy of cancer patients, and investigate their association with CAM use.

## Methods

### Participants

The questionnaire was distributed to patients of the outpatient oncology department at “Jena University Hospital” and the rehabilitation facility “Paracelsus-Klinik am See”, between September and November 2018. The patients were informed that participation in the survey will be anonymous.

For this study, we collected the data from 308 patients. The information regarding the demographic data was not completely filled in for one-third of the questionnaires. However, the data on other predictors for CAM use were included in regression analysis.

### Questionnaires

The survey was a composition of six sections, one on personal data (age, gender, education), four questionnaires investigating personality properties and one questionnaire on CAM use.

To measure resilience, the RS 11, a reliable short version of the RS-25 questionnaire was used (Schumacher et al. 2005), reliability: *α* = 0.86 (von Eisenhart Rothe et al. 2013). The resilience is defined as a protective personality property which has been positively correlated with healthy adaptation. Patients were supposed to rate how much their usual behavior applied to the given statements, concerning belief in their own abilities (Likert scale 1 = “doesn’t apply at all” to 7 = “fully applies”). Studies of the questionnaire show a positive correlation with well-being, and a negative correlation with tendencies towards mood disorders (von Eisenhart Rothe et al. 2013).

The ASKU (Allgemeine Selbstwirksamkeit Kurzskala, English: Short Scale for Measuring General Self-efficacy Beliefs) is a three-item scale by Beierlein et al. (Beierlein et al. 2012). It is a self-assessment instrument, used to investigate subjectively perceived expectation to personal competence to resolve difficulties in everyday life and to cope with critical situations. The items were measured on a 5-point Likert scale (Likert scale 1 = “doesn’t apply at all” to 5 = “fully applies”). Reliability and validity were found to be sufficient with a reliability of *ω* = 0.81 to *ω* = 0.86 tested in two samples (Beierlein et al. 2012). The TPV (Transpersonelles Vertrauen, English: transpersonal trust) is a valid and reliable instrument to assess patients’ spiritual and religious concepts using a 3-point Likert scale (Likert scale 0 = “doesn’t apply at all” to 3 = “fully applies”), and consisting of 11 items. Reliability was tested to variate from *α* = 0.89 to *α* = 0.95 (Albani et al. 2002). The FFA-14 (Freiburger Fragebogen für Achtsamkeit, English: Freiburg questionnaire for mindfulness) asks patients to rate statements about inner attitudes towards themselves. We used a short version of the originally 30-item survey. This version has been shown to measure the construct of mindfulness, independently from pre-existing theoretical knowledge about meditation or Buddhist philosophy on a 4-point Likert scale (Likert scale 1 = “doesn’t apply at all” to 4 = “fully applies”). A test for reliability showed a Cronbachs *α* of *α* = 0.93 (Walach et al. 2004).

The 5th section originally consisted of the CAM-questionnaire developed by the working group Prevention and Integrative Oncology of the German Cancer Society (Huebner et al. 2014). To simplify, we shortened this section focusing on the questions if patients were interested in CAM, for what reasons and if the participants had used CAM during the past three months. Finally, a list of current complementary medicine therapy options and nutritional supplements was prepared. The patients were asked to indicate whether they had used the method in the past three months. The CAM section consisted of closed questions that could only be answered with “yes”, “no” or “I am not sure” and questions with multiple possible answers and the option to add own experiences in an open text field.

### Statistical analyses

Analyses were conducted in SPSS (version 25). Binary logistic regressions were run to test if sociodemographic variables (age, gender and education), resilience, self-efficacy, spirituality, transpersonal confidence and mindfulness were associated with the dependent variable CAM use (use vs. no use). To differentiate more precisely within the group of CAM users, two dependent variables for CAM use were created, one called “CAM use—biological-based methods”, the other “CAM use—holistic and mind–body methods”. The variable “CAM use—biological-based methods” contains complementary methods which are also frequently prescribed by conventional medical practitioners, such as vitamin b, c, d and e and trace elements, such as zinc and selenium. The variable “CAM use—holistic and mind–body methods” includes methods that would most likely not be prescribed by conventional medical practitioners. We categorized the use of medicinal plants, such as mistletoe, Chinese medicine, such as acupuncture and tees, prayer, meditation, yoga and other relaxations methods, homeopathy, consultation of a healer, the use of amygdalin (“vitamin b17”) and various dietary methods into this variable. Two regression models were run, one for each dependent CAM variable. To assess the predictive value of the predictors, Odds Ratios (OR) were calculated in the logistic regression. An OR above one indicates a positive relationship between the predictor and the dependent variable. An OR below 1 implies a negative association.

Tests for multicollinearity were run in the logistic regressions. The VIF (variance inflation factor) ranged between 1.10 and 1.51 indicating that multicollinearity was not an issue (Ziegel and Myers 1991). Outliers and influential cases could not be detected using Cook’s distance, standardized DFBetas and standardized residuals.

#### Ethical vote

The survey was approved by the “Ethic Committee of Jena University Hospital of Friedrich-Schiller-University Jena”.

## Results

### Demographic data

For this study, we collected the data from 308 patients. Of these 51.1% (*N* = 101), participants were female, 48.5% (*N* = 95) male. The biggest part of participants (55.9%, *N* = 114) belonged to the age group of the 50- to 70-year-olds. A detailed overview of the characteristics of the study sample can be found in Table 1.

**Table 1:** Demographic data (*N* = 308)

Characteristics	*N* (%)
Age in years	
Younger than 50	25 (12.3)
50–70	114 (55.9)
Older than 70	65 (31.9)
Gender	
Women	101 (51.5)
Men	95 (48.5)
Education	
Basic	86 (44.3)
Middle	53 (27.3)
High	55 (28.4)

### Interest in CAM and CAM use

Among the 55.9% (*n* = 160) of the patients who indicated they were interested in CAM, 48.1% (*n* = 77) stated to only having developed that interest since the diagnosis of their cancer disease. 47.5% of the participants stated to have used CAM in the past months. They were asked to specify the applied methods. The most common practice used by the study population was food supplements. 31.8% of the CAM users stated to have taken nutritional supplements, which we categorized as biological-based methods. The second most frequently applied practice, with 10.8%, was praying, followed by homeopathy with 10.5%, we categorized both of those methods as holistic and mind–body.

Table 2 shows a detailed overview of the frequency of occurrence.

**Table 2:** Model parameters for CAM use, CAM methods applied (*N* = 308)

Model parameters	Yes* n* (%)	No *n* (%)	Uncertain *n* (%)
Interest in CAM	160 (55.9)	55 (19.2)	71 (24.8)
CAM use	135 (47.5)	127 (44.7)	21 (6.8)
Food supplements	97 (31.8)	208 (68.2)	
Mistletoe	12 (3.9)	293 (96.1)	
Medicinal plants	26 (8.5)	279 (91.5)	
Acupuncture	12 (3.9)	293 (96.1)	
Chinese tees	13 (4.3)	292 (95.7)	
Homeopathy	32 (10.5)	273 (89.5)	
Prayer	33 (10.8)	272 (89.2)	
Yoga/Thai chi/qi gong	25 (8.2)	280 (91.8)	
Meditation	24 (7.9)	281 (92.1)	
Relaxation methods	26 (8.5)	279 (91.5)	
Consultation at a healer	3 (1.0)	302 (99.0)	
Amygdalin	3 (1.0)	302 (99.0)	
Ketogenic diet	16 (5.2)	289 (94.8)	
Vegan diet	5 (1.6)	300 (98.4)	
Others	25 (8.2)	279 (91.8)	

### Predictors for CAM use “biological-based methods”

The first regression aimed to identify predictors for the use of “biological-based CAM methods”. In this model, the effect of female gender was not a significant predictor for CAM use. Nevertheless, the results showed an increased tendency to use CAM methods by women (OR = 0.453, CI 0.190–1.079, *p* = 0.074). Patients with lower education used significantly less biological-based methods of complementary medicine than patients with higher education (OR = 0.238, CI 0.083–0.685, *p* = 0.008). The effect was weaker when comparing patients with mid-level education with participants with higher education (OR = 0.622, CI 0.221–1.757, *p* = 0.371). Patients of the lowest age group (younger than 50) used significantly more biological-based methods of CAM than the older participants (50–70 years) (OR = 6. 080, CI 1.636–22.599, *p* = 0.007). When comparing participants of the age group 50–70 with the oldest age group (older than 70 years), there was no effect on the use of CAM detectable (OR = 0.908, CI 0.323–2.547, *p* = 0.854). Of the other independent variables, only attentiveness showed a significant effect on the use of conventional methods of CAM (OR = 1.079, CI 1.001–1.163, *p* = 0.047). None of the other traits could be linked to CAM usage in the regression model (Table 3).

**Table 3:** Model parameters for CAM use—“biological-based methods” (*N *= 308)

Model parameters	OR	CI	*p*
Constant	1.798		.728
Women vs. Men	0.453	0.190–1.079	.074
Education higher vs middle	0.622	0.221–1.757	.371
Education higher vs basic	0.238	0.083–0.685	.008
Age 50–70 vs. Younger than 50	6.080	1.636–22.599	.007
Age 50–70 vs. Older than 70	0.908	0.323–2.547	.854
Resilience	0.981	0.934–1.030	.436
Attentiveness	1.079	1.001–1.163	.047
Self-efficacy	0.569	0.228–1.123	.104
Spirituality	1.027	0.979–1.078	.275
Chi² (*df*)	7.437 (8)		

### Predictors for CAM use “holistic and mind–body methods”

The aim of the second regression model was to identify predictors for the use of holistic and mind–body methods of CAM. This time, female gender was a significant predictor. Women used significantly more holistic and mind–body methods of CAM than men (OR = 0.363, CI 0.153–0.864, *p* = 0.022). Also, higher education compared to basic education showed a significant effect on the use of holistic and mind–body methods of CAM. Patients with higher education stated to have significantly higher use of holistic and mind–body methods of CAM than patients with a lower level of education (OR = 0.242, CI 0.085–0.691, *p* = 0.008). Again, when comparing levels of CAM use within the group of participants with middle and higher education, no effect was detectable (OR = 0.995, CI 0.344–2.876, *p* = 0.993). Age was not a significant predictor in this regression model. However, a non-significant effect, contrary to results of the first regression, can be deduced out of the results, indicating that participants older than 70 years have higher levels of holistic CAM use than the younger participants (OR = 0.395, CI 0.143–1.090, *p* = 0.073). In this regression, another strongly significant predictor for the use of holistic and mind–body methods was spirituality (OR = 1.125, CI 1.064–1.189, *p* = 0.000). None of the other predictors were significant in the second model as shown in Table 4.

**Table 4:** Model parameters for CAM use—“holistic and mind–body methods” (*N* = 308)

Model parameters	OR	CI	*P*
Constant	0.449		.631
Women vs. Men	0.363	0.153–0.864	.022
Education higher vs middle	0.995	0.344–2.876	.993
Education higher vs basic	0.242	0.085–0.691	.008
Age 50–70 vs. Younger than 50	2.223	0.593–8.339	.236
Age 50–70 vs. Older than 70	0.395	0.143–1.090	.073
Resilience	0.995	0.947–1.046	.849
Attentiveness	1.023	0.951–1.100	.568
Self-efficacy	1.057	0.532–2.100	.874
Spirituality	1.125	1.064–1.189	.000
Chi² (*df*)	7.047 (8)		

## Discussion

This study investigated sociodemographic variables and personal traits as predictors for CAM use. By differentiating within the complementary methods and dividing them into the groups “holistic and mind–body methods” and “biological-based methods”, different predictors were identified.

The percentage of CAM users was 47.9%. This number is comparable with the results of other German studies (Micke et al. 2009; Dubois et al. 2019).

### Applied methods in the study population

Confirming earlier reports, food supplements, such as vitamin B, C, D, E, selenium and zinc, were the most frequently applied methods in the study population (Sparber et al. 2000; Molassiotis et al. 2005; Huebner et al. 2014). The stated percentage share of use was also in line with previous research.

Furthermore, prayer, sports, such as Yoga, meditation and relaxation methods, were reported to be popular CAM methods, respectively, used by approximately 10% of the participants. Those numbers also reflect the results of previous studies (Micke et al. 2009; Huebner et al. 2014). As these non-biologically based methods do not interfere with conventional cancer therapies, they could and should be supported by physicians. Contrary to this, methods like medicinal plants, which were also popular among the study population with 8. 5% indicated use, can have side effects and provoke interactions when not discussed with the physician.

### Sociodemographic variables as predictors

By establishing two groups of CAM methods, different predictors could be identified. Concerning well-established sociodemographic predictors for CAM use, the study showed consistent results with various other studies that had investigated CAM use in the past (Richardson et al. 2000; Dubois et al. 2019; Frass et al. 2012). Younger age and higher education showed and significant association with the use of biological-based methods of CAM, female gender was not a significant predictor in this regression, but a positive correlation was detectable. Within the group of holistic and mind–body methods, female gender and higher education were identified as significant. Even though age was not a significant predictor in this group, the relation between CAM use and younger age, was positive. Characteristics like higher education and younger age might indicate better access to information about complementary methods or even a higher knowledge about the disease and possible therapy options. Apart from that, higher education is associated with higher economic status, which offers the possibility of using methods, and task alternative healers that might not be covered by health insurance.

Another possible explanation for higher CAM use in young patients might be a higher level of social integration and thus more support from others, who might have made positive experiences with complementary methods.

### Personal traits

#### Spirituality

It seems likely to assume that people with high levels of spirituality are interested and possibly more open to alternative therapies, seeking to satisfy their spiritual needs. One possible explanation for such an interest might be the correlation of spirituality with an active coping style (Holland et al. 1999). Choosing an alternative method and applying it might give patients a sense of control and active participation in the process of healing. Also, spiritual needs are often not met by conventional medicine, nor do patients have a religious community to find support (Balboni et al. 2007). However, CAM methods seem to give appropriate significance to the psychological aspect of healing and the spiritual needs of cancer patients (Hsiao et al. 2008).

﻿In this study, spirituality could be identified as a strong predictor for, what we defined as “holistic and mind–body methods” of CAM. Concerning the conventional methods, such as the use of vitamins and other food supplements, no positive relation with spirituality could be found. Other studies have identified spirituality, when differentiated from religiosity, as a predictor for both biological-based and holistic and mind–body methods (Hsiao et al. 2008; Smith et al. 2008). While at a first glance, this seems to be a contradiction to our data, the most probable explanation is that the TPV instrument is measuring the personal relation to Holy Spirit and not other dimensions of spirituality. In fact, the needs of patients with a high level of piety as measured in our study might well be only satisfied by mind–body methods, while other dimensions might be more related to social activities or a sense of responsibility for oneself which may entail use of social contacts or biological-based CAM methods.

#### Attentiveness

Attentiveness was identified as another significant predictor for CAM usage. The concept of mindfulness and attentiveness has been shown in various studies to promote health and well-being (Baer 2003; Brown and Ryan 2003; Baltzell and Cote 2017).

Techniques, such as Yoga, meditation and other methods, with Buddhist origin have been promoting mindfulness, as an essential component of their practice, for centuries (Baltzell and Cote 2017). Relatively to that, western society has only recently discovered the benefits of mindfulness training. Literature is emerging in that aspect and plenty of definitions of attentiveness are developing. Not only can it be trained but there are individuals with higher tendencies of self-care, and self-awareness. If seen as a character-trade or personality property, mindfulness can be measured and has been identified as a predictor for social behaviors (Lakey et al. 2008; Ruedy and Schweitzer 2010). However, research on how mindfulness as a personality property affects decision-making in disease and the use of CAM is sparse. We identified attentiveness as a significant predictor for the use of biological-based methods of CAM, such as vitamins, or other food supplements like selenium and zinc, but not as a predictor for what we defined as “holistic and mind–body methods”, e.g. Buddhist methods like Yoga, Thai Chi, Qi Gong. This is interesting when considering the origins of mindfulness trainings. However, the results go in line with the assumption that mindfulness promotes health. People with higher levels of mindfulness might have more significant interest in their body functions, and a greater knowledge about what strengthens their body and mind. It can be assumed that they have greater recourses, and capacities to inform themselves about possible treatment options. Furthermore our findings could go in accordance with a study published in 2008, indicating that higher levels of mindfulness promote a less defensive and more open communication style (Lakey et al. 2008). This could enable patients to communicate openly with their physician about possible alternative treatment options and thus explain the patients’ choice of physician-approved alternative methods.

#### Self-efficacy and resilience

It is evident to assume that in line with attentiveness also other personal traits like self-efficacy and resilience promote interest in self-care and participation in a healing process. It has been established in various studies in the past that resilience and self-efficacy positively relate to positive health behavior and well-being (Strecher et al. 1986; Cosco et al. 2016). However, in this study, we were not able to prove a significant relationship between these two personal traits and the use of CAM. Our findings do go in line with a former study from our group (Ebel et al. 2015). Equally to our study, self-efficacy did not show a significant effect on CAM use. Yet research in this field is still very sparse, further research with a bigger study sample might be needed to validate our results and to find explanations for them.

### Limitations

Our results were collected in a rehabilitation center, this might represent a special group of patients that have been living with their diagnosis for a while and have had time to reflect upon personal coping methods, e.g. the use of CAM.

Another point that could be considered a limitation to the work might be the fact that the “TPV”, which is the instrument we used to measure spirituality within our study population, does only measure one dimension of spirituality. It evaluates spirituality in the sense of religion, the relation to the Holy Spirit. More studies are needed to learn more on the influence of all dimensions of spirituality including altruism, love, awe and gratitude on the needs and coping of cancer patients in the health care system and to develop support methods addressing these needs more differentiated.

Another limitation point might be the missing differentiation between the tumor types. Furthermore, there might be systemic differences between participants and patients who decided not to participate, as participation was voluntary.

### Conclusion

This study is among the first to take a more detailed look at how numerous personal traits relate with the use of CAM methods and differentiate between the applied methods. We showed that next to sociodemographic predictors, like age, sex, and education, also personal traits anticipate the use of CAM. Furthermore, it demonstrates that within the group of CAM users, there are unambiguous differences between the participants of the study. While the use of “holistic and mind–body methods” is associated with higher levels of spirituality, a predictor for “biological-based methods” is attentiveness.

Our finding should be considered by conventional health care providers and could be integrated into a holistic assessment, to offer information about complementary medicine and meeting patients’ needs. Physicians may need to improve their understanding of personal traits influencing the use of CAM methods, and therefore decision-making and health behavior. This might ease the way into more open communication, between patients and physicians, build mutual confidence and potentially facilitate patients’ decisions in using health-wise viable CAM methods.
